# Random forest algorithm for predicting postoperative delirium in older patients

**DOI:** 10.3389/fneur.2023.1325941

**Published:** 2024-01-11

**Authors:** Weixuan Sheng, Xianshi Tang, Xiaoyun Hu, Pengfei Liu, Lei Liu, Huihui Miao, Dongxin Wang, Tianzuo Li

**Affiliations:** ^1^Department of Anesthesiology, Beijing Shijitan Hospital, Capital Medical University, Beijing, China; ^2^Key Laboratory of National Health Commission on Parasitic Disease Control and Prevention, Key Laboratory of Jiangsu Province on Parasite and Vector Control Technology, Jiangsu Institute of Parasitic Diseases, Wuxi, China; ^3^Department of Science and Technology, Beijing Shijitan Hospital, Capital Medical University, Beijing, China; ^4^Department of Anesthesiology, Peking University First Hospital, Beijing, China

**Keywords:** postoperative delirium, random forest, confusion matrix, partial dependence graph, older patient

## Abstract

**Objective:**

In this study, we were aimed to identify important variables via machine learning algorithms and predict postoperative delirium (POD) occurrence in older patients.

**Methods:**

This study was to make the secondary analysis of data from a randomized controlled trial. The Boruta function was used to screen relevant basic characteristic variables. Four models including Logistic Regression (LR), K-Nearest Neighbor (KNN), the Classification and Regression Tree (CART), and Random Forest (RF) were established from the data set using repeated cross validation, hyper-parameter optimization, and Smote technique (Synthetic minority over-sampling technique, Smote), with the calculation of confusion matrix parameters and the plotting of Receiver operating characteristic curve (ROC), Precision recall curve (PRC), and partial dependence graph for further analysis and evaluation.

**Results:**

The basic characteristic variables resulting from Boruta screening included grouping, preoperative Mini-Mental State Examination(MMSE), CHARLSON score, preoperative HCT, preoperative serum creatinine, intraoperative bleeding volume, intraoperative urine volume, anesthesia duration, operation duration, postoperative morphine dosage, intensive care unit (ICU) duration, tracheal intubation duration, and 7-day postoperative rest and move pain score (median and max; VAS-Rest-M, VAS-Move-M, VAS-Rest-Max, and VAS-Move-Max). And Random Forest (RF) showed the best performance in the testing set among the 4 models with Accuracy: 0.9878; Matthews correlation coefficient (MCC): 0.8763; Area under ROC curve (AUC-ROC): 1.0; Area under the PRC Curve (AUC-PRC): 1.0.

**Conclusion:**

A high-performance algorithm was established and verified in this study demonstrating the degree of POD risk changes in perioperative elderly patients. And the major risk factors for the development of POD were CREA and VAS-Move-Max.

## 1 Background

Postoperative delirium (POD) refers to delirium that occurs after surgery and is defined as an acute mental disorder characterized by disturbances of consciousness, attention, and cognition. The reported incidence ranges from 5 to 52% in elderly patients (1). The mechanism of delirium is not totally clear, but is generally believed to be a result of co-action of predisposing factors and external stress. However, its highly preventable nature determines that early and effective intervention would reduce its occurrence and related treatment costs which are estimated to exceed 100 billion US dollars (2) annually across the globe. Studies have shown that early preventive measures decreased the odds of POD by about 30% in high risk patients (3, 4). Therefore, it is crucial to identify and control the relevant factors contributing to the POD development.

As a major branch of artificial intelligence (AI), machine learning has the advantages in establishing models with more stable and accurate prediction, and is therefore increasing used for such purposes as clinical prediction. The use of artificial intelligence to solve clinical problems and the construction of a precision medical research model based on complex data acquisition and integration utilization will drive innovation and development in clinical medicine. In this study, based on the reviewed database of elderly patients, we used machine learning algorithms to screen risk factors and to predict the risk of POD, in order to assist clinicians in developing personalized management plans for patients in a timely manner.

## 2 Materials and methods

### 2.1 Study design and subjects

The data in this article came from delirium in Older Patients after Combined Epidural-General Anesthesia or General Anesthesia for Major Surgery: a Randomized Trial (5). This was a secondary analysis of database from a previous trial. The trial protocol was approved by the Institutional Review Committee of Peking University (Approval No. 00001052-11048) and the ethics committees of five participating centers, and was registered with the Chinese Clinical Trial Registry (www.chictr.org.cn; identifier: ChiCTR-TRC-09000543) and ClinicalTrials.gov (identifier: NCT01661907). The original trial was conducted in five tertiary hospitals in Beijing, China. All participants provided written informed consent.

During the original trial, we enrolled patients aged 60–90 years who underwent elective non-cardiac thoracic or abdominal surgery for at least 2 h and required patient-controlled analgesia after surgery. We excluded those who had severe nervous system disease, acute myocardial infarction or stroke, severe cardiac insufficiency, severe hepatic insufficiency or renal failure within 3 months, or any contraindication to epidural anesthesia.

### 2.2 Anesthesia and perioperative care

No premedication was given. Patients were randomized to receive either general anesthesia or combined epidural-general anesthesia in the original trial. For those assigned to general anesthesia alone, patient-controlled intravenous analgesia was provided after surgery. For those assigned to combined anesthesia, epidural block was performed during surgery, followed by patient-controlled epidural analgesia after surgery. Other perioperative care including adverse events were managed per routine.

### 2.3 Data collection and outcome assessment

Baseline data included demographic characteristics, preoperative comorbidity, surgical diagnosis, and main laboratory test results. General health status was evaluated with the Charlson Comorbidity Index and ASA physical status classification. Cognitive function was evaluated with the Mini-Mental State Examination (MMSE). Anxiety and depression were evaluated with the Hospital Anxiety and Depression Scale (HAD). Intraoperative data included type and duration of anesthesia, type and dose of medications, circulation parameters, and type and duration of surgery.

After surgery, patients were followed up twice daily during the first 7 days, and then weekly until 30 days. Pain severity was assessed with the Numeric Rating Scale during the first 7 postoperative days. Delirium assessment: delirium for patients in ICU was assessed by the Confusion Assessment Method for the ICU (CAM-ICU) (6), which has been validated in Chinese patients in the ICU setting (7) and the feasibility of which had been established in our prior studies (8, 9). For patients did not admitted to ICU, delirium was assessed by CAM.

Fifty-eight potentially useful characteristics considered in this study included the followings: basic personal information including age and gender; preoperative comorbidities; Charlson Comorbidities Index scores; MMSE and Hospital Anxiety and Depression Scale (HAD) score; preoperative laboratory examination results; intraoperative anesthesia medication, circulation parameters and grouping (simple general anesthesia group and general anesthesia combined with epidural anesthesia group); postoperative NRS, worst APACHE II score, etc.

### 2.4 Statistical analysis and sample size

R (version 4.2.2) and RStudio (version 2023.06.0 + 421) were used for data statistical analysis. The normal distribution of numeric variables was tested by the Shapiro–Wilk test. Continuous variables with a normal distribution were presented as mean ± standard deviation (SD) and compared using the independent-sample *t*-test. Continuous variables with non-normal distribution were presented as median (IQR) and compared using Mann–Whitney U test. Categorical data were expressed as number (%) and analyzed using the chi-square test or Fisher’s exact probability test. Parameters with missing data of more than 20% were excluded from the final dataset. Parameters with missing data of less than 20% were interpolated using the missForest package. MissForest package is a non-parametric method that utilizes random forests to impute missing values, suitable for both continuous and categorical variables. Its core algorithm is to use known variables as independent variables and variables containing missing values as dependent variables to establish a random forest to predict missing values. It yields an out-of-bag (OOB) imputation error estimate.

Independent influencing factors were derived from POD related important characteristic variables used for Boruta screening. Creates possibly balanced samples by Smote technique (the synthetic minority over-sampling technique, Smote) (10–12). The basic idea of Smote technique is to analyze minority samples and manually synthesize new samples based on minority samples to add to the dataset.

The selection of model hyperparameter optimization used repeated *k*-fold cross validation (folds = 10, repeats = 10) on data set. Repetitive *k*-fold cross validation is an extension of *k*-fold cross validation. In this article, it divides the dataset into 10 mutually exclusive subsets of the same size. Each subset is used as a validation dataset to validate the model, while the other nine subsets are used as training datasets to train the model. The appeal process is repeated 10 times. Meanwhile, the performance of the machine model is directly related to hyperparameters. The better the hyperparameter tuning, the better the resulting model.

The mlr3verse package was used to successively build models from the data set via repeated *k*-fold cross-validation, hyperparameter optimization, and Smote technique 4 machine learning prediction models (LR, KNN, CART, and RF) were included and analyzed, with RF proven better than the other 3 in terms of misclassification rate. RF as the optimal model is further analyzed and evaluated by calculating the parameters of confusion matrix and drawing ROC and PRC. The iml and DALEX package was used to draw importance ranking for important characteristic variables, partial dependence graph, and break down profile to interpret the optimal model.

Basic Principles of Random Forest:


$$Y=H\left(x\right)=\arg\max_{y}\overset{n}{\underset{k=1}{\sum }}I\left(h_{k}\left(x\right)=y\right)$$

*H*(*x*) is a combination classification model; *Y* is the final classification result; $h_{k}$ (*x*) is a single decision tree classifier; *y* is the classification result of a single decision tree classifier; *I* (·) is an indicative function.

For the binary classification prediction model, the calculated final sample size obtained through the pmsampsize function of RStudio was 1,459, less than the sample size 1,720 included in this study, with function parameters set as follows: the adjusted maximum R2: 0.327; The number of independent variable parameters to be included: 30; Incidence of postoperative delirium: 0.05 (13–15).

## 3 Results

### 3.1 Flow chart and baseline of clinical data

**Figure 1:**
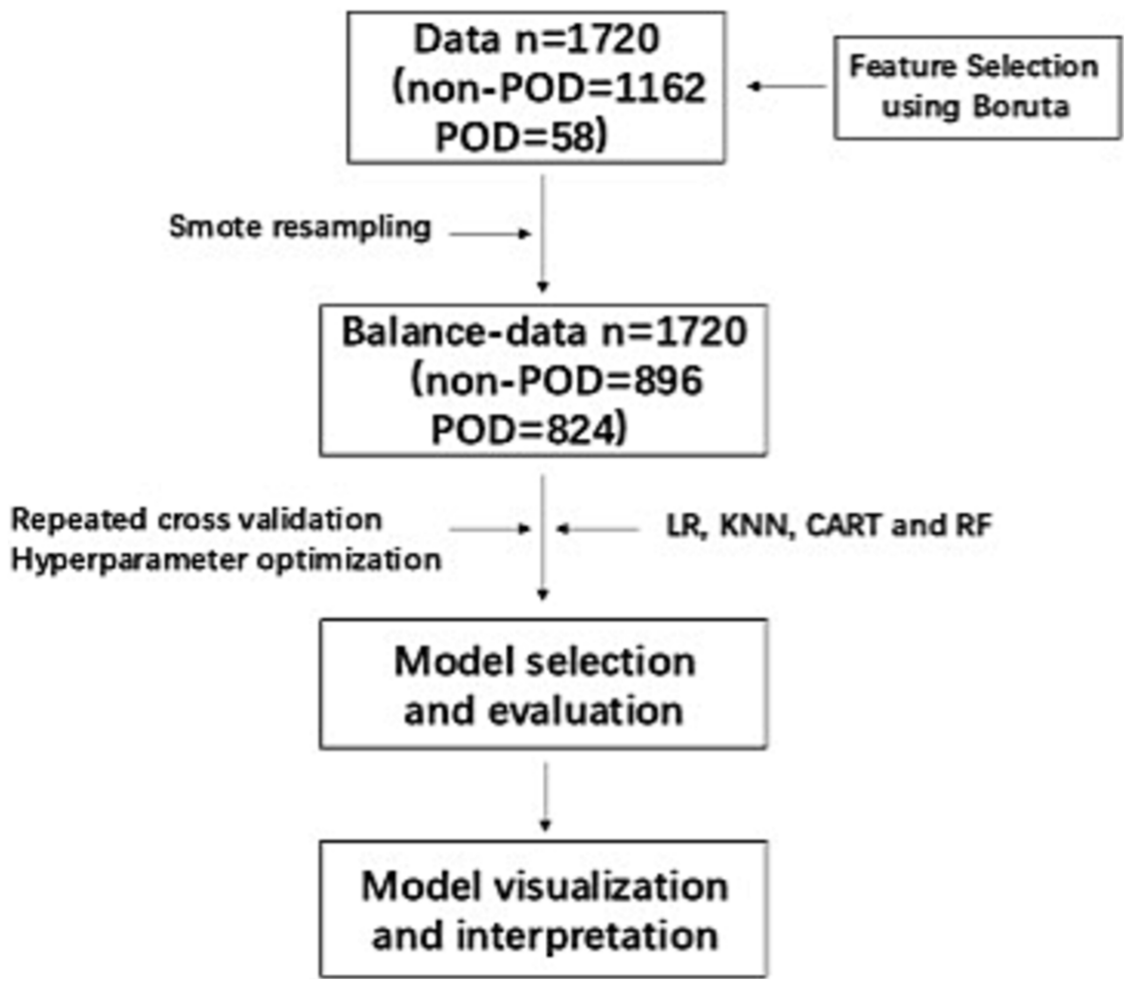
Flow chart of clinical data.

A total of 1720 patients were included in the original data. The process of data inclusion, model establishment, and evaluation were presented in Figure 1. First, Boruta was applied to filter characteristic variables in the original data. Then, Smote technology was used to establish a balanced-data (*n* = 1720, non-POD = 896, POD = 824) on top of the original data. Finally, repeated *k*-fold cross-validation and hyperparameter optimization were used to obtain the optimal model in balance-data, and to perform validation and interpretation.

Among the included patients, 58 (3.372%) developed delirium within the first 7 postoperative days. Comparison of preoperative, intraoperative, and postoperative variables between POD and non-POD patients were listed in Supplementary Table 1. The CREA was significantly lower in POD group than non-POD group [73.50 (61.00–93.00) vs. 86.00 (76.00–99.00), *p* < 0.001]. The depression score was significantly higher in patients with POD. The patients with POD had lower nitrosoxide (%), postoperative morphine (mg), GEA-PCEA (%) and higher seveflurance (%), midazolam (mg), urine (mL), bleeding (mL), MAP (mmHg), MHR (times/min), perioperative morphine (mg), ICU duration (min), intubation duration (min), APACHE-II, VAS-Rest-M, VAS-Move-M, VAS-Rest-Max, VAS-Move-Max, VAS-Rest-Min, VAS-Move-Min, GA-PCIA (%), and intraoperative hypotension (%).

### 3.2 Screening of characteristic variables using Boruta

**Figure 2:**
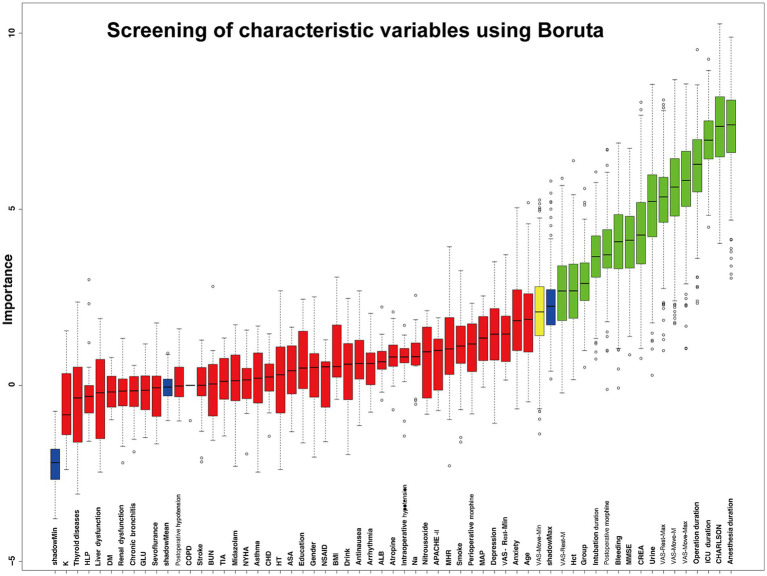
Screening of characteristic variables using Boruta.

Boruta analysis showed that grouping, preoperative MMSE, CHARLSON, preoperative HCT, preoperative serum creatinine, intraoperative bleeding volume, intraoperative urine volume, anesthesia duration, operation duration, postoperative morphine dosage, ICU admission, VAS-Rest-M, VAS-Move-M, VAS-Rest-Max, and VAS-Move-Max were 16 characteristic variables included in the model in Figure 2.

### 3.3 Model establishment, selection, and evaluation

**Figure 3:**
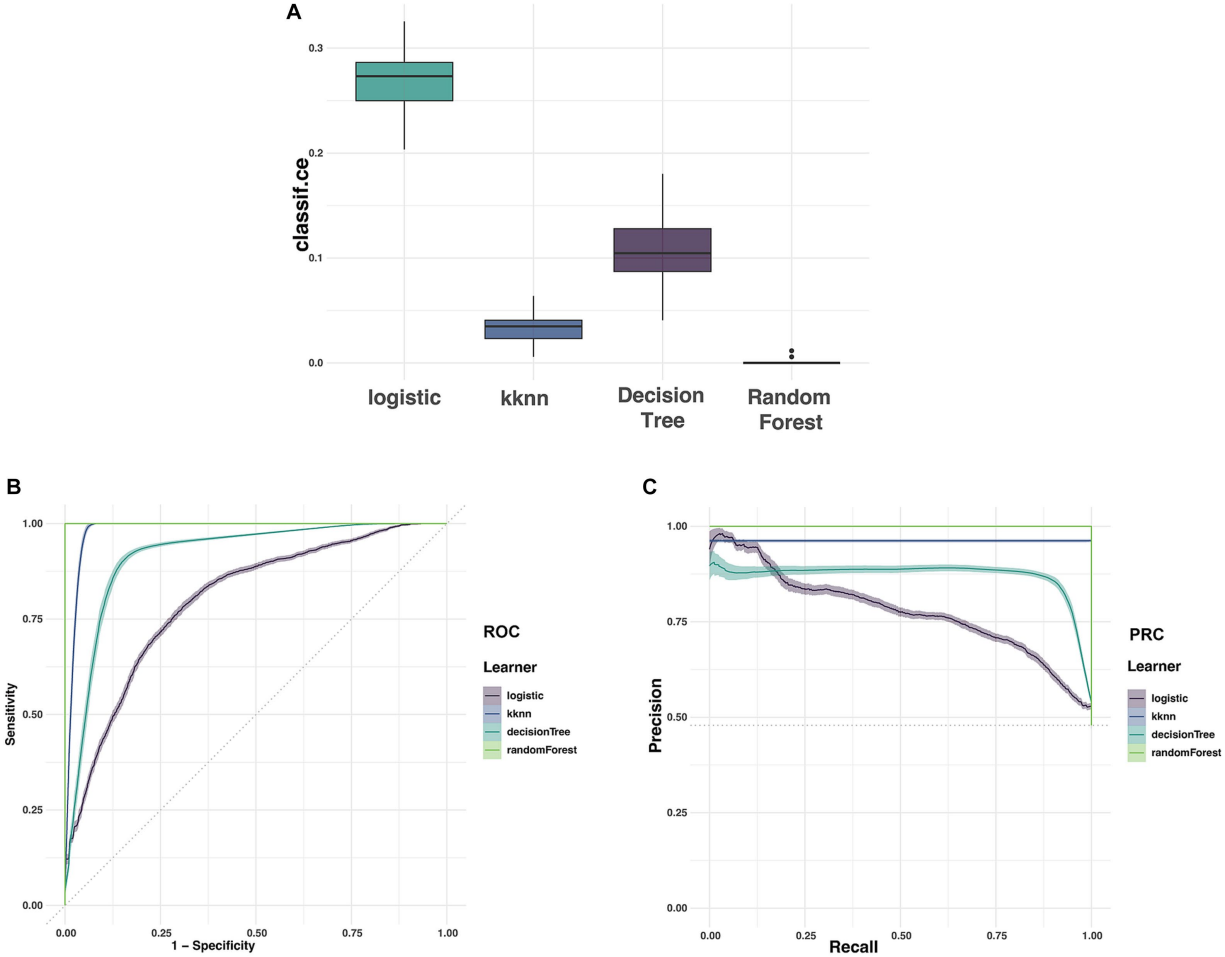
The Ce, ROC, and PRC of the clinical data (**A** represented Ce of four models; **B** represented ROC of four models; **C** represented PRC of four models).

After identifying these 16 variables, machine learning models were used to predict POD. CE, AU-ROC, and AUC-PRC were important indicators used to evaluate prediction models. Among the four models established, RF showed the best performance in error rate (Ce), ROC, and PRC from Figure 3. From Figure 3A, it can be determined that Ce of RF is the lowest among the four models. From Figures 3B,C, it can be determined that the AUC-ROC and AUC-PRC of RF are the highest among the four models. Finally, we calculated the parameters of confusion matrix in RF: Accuracy 0.998, MCC 0.997, AUC-ROC 1.0, and AUC-PRC 1.0. The above data showed that the random forest model had excellent performance in accuracy, overall performance, overall discrimination, and positive result discrimination.

### 3.4 Importance ranking and partial dependency graph of characteristic variables

**Figure 4:**
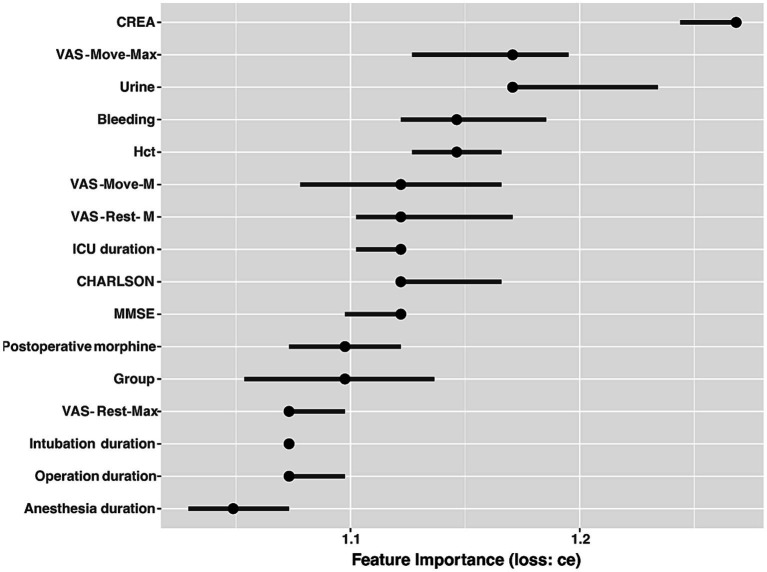
Importance ranking of characteristic variables.

**Figure 5:**
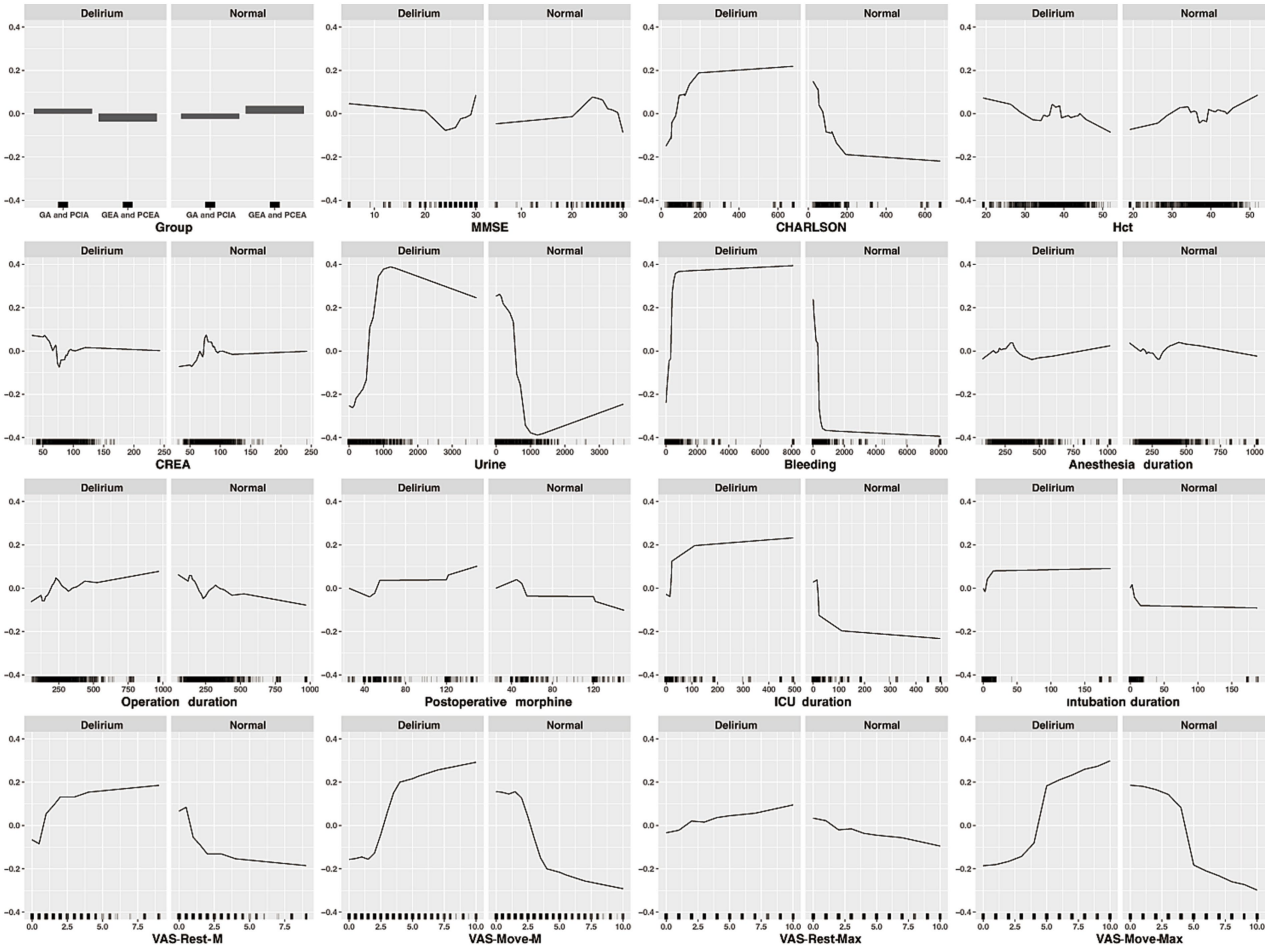
Partial dependency graph of characteristic variables.

Ranking and partial dependency graphs of 16 characteristic variables were established through RF model in Figures 4, 5. In importance ranking, it could be intuitively seen how much each characteristic variable contributes to the predicted variable. In our study, the level of CREA and VAS-Move-Max ranked first and second in importance. Partial dependency graph was used to analyze RF model, showing the reflect the influence of each feature in the sample and also showing the positive and negative influences. At the same time, when the characteristic variable was above or below the cutoff value, the predictive variable would undergo a qualitative transformation.

### 3.5 Break down profile to explain a single sample in RF

**Figure 6:**
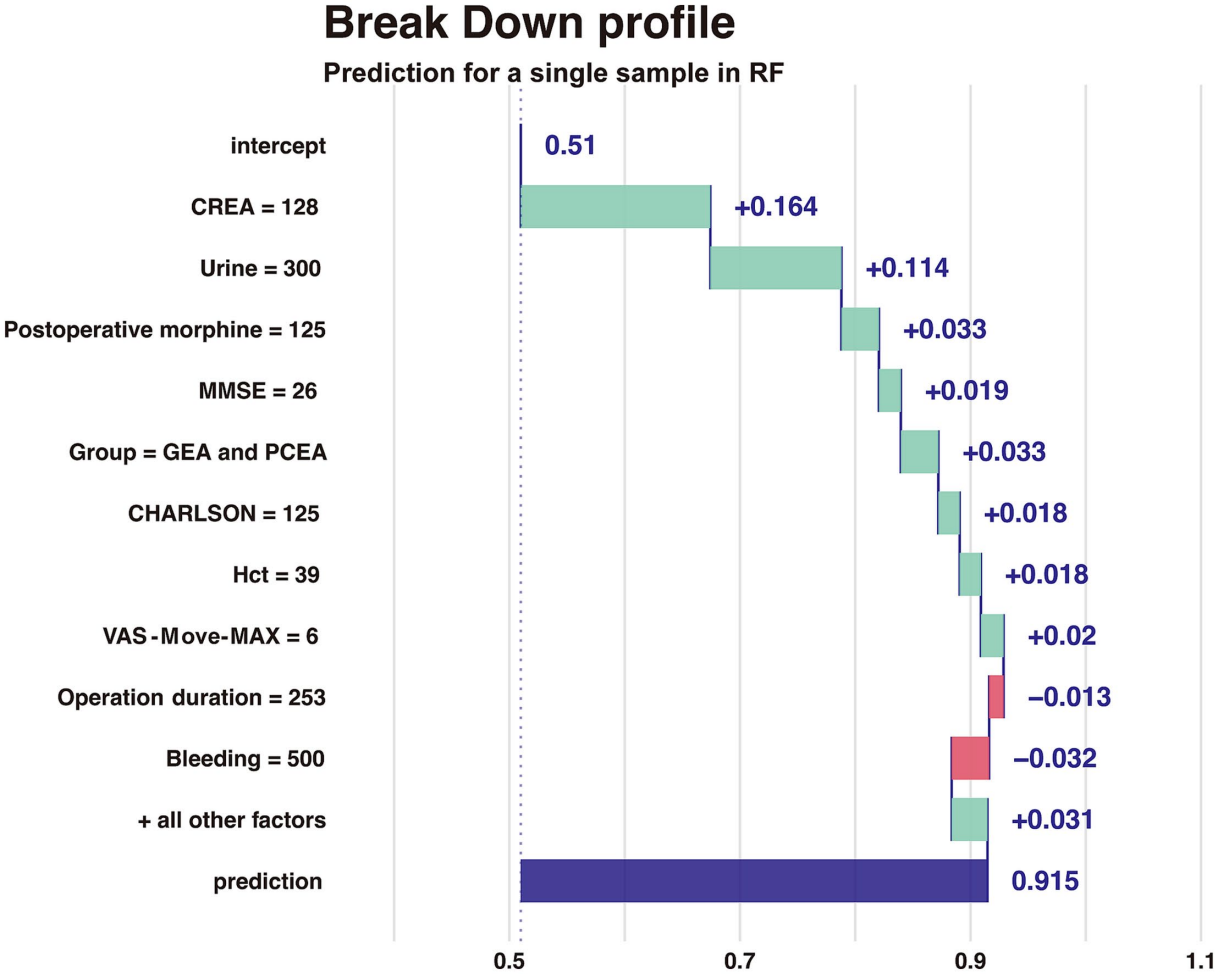
Break down profile of RF.

The Breakdown profile visualizes the contribution of each variable to the prediction for a single sample in Figure 6. The model predicts that the value of a sample (delirium as an outcome variable) is 0.915, and the red or blue bars display the impact of each variable on the prediction. The predicted value is equal to the sum of the contributions of each feature.

## 4 Discussion

In importance ranking, it can be intuitively seen that Creatinine, as a feature variable, has the greatest contribution to the predictive variable. Furthermore, through partial dependency graphs, POD occurrence probability was increased no matter when preoperative creatinine levels were lower or higher than normal, which is consistent with the published results (16), demonstrating that kidney function has an impact on brain cognitive ability. The possible reason is that metabolic disorders of renal function (abnormal levels of creatinine, etc.) affect the cognitive function.

As the influencing factors before surgery, the partial dependence graph of preoperative MMSE used for cognitive function assessment showed that patients with MMSE<20 and diagnosed with moderate to severe cognitive impairment before surgery would have the significantly increased probability of POD occurrence, confirming studies reporting the correlation between preoperative MMSE and POD (17) and identifying the preexisting cognitive impairment as the important basis of POD. This study showed that CHARLSON score was positively correlated with postoperative cognitive impairment, with the cutoff value of 100 in the partial dependence graph, which is consistent with former literature reports (18) and possibly related to the stress state caused by the existing physical illness of patients.

As POD predictors (19, 20) directly related to cerebral hypoxia, when preoperative HCT < 30, the risk of POD increased with the decrease of HCT, and when the intraoperative blood loss was less than 500 mL, the curve of the partial dependence graph rose sharply, and then at a gentle pace. The model also predicted that the increased probability of POD would come with the increase of urine volume, with the volume of 500 mL as the visible cutoff value of the curve which rose sharply when urine volume was between 500 and 1,000 mL and fell slowly when urine volume was over 1,000 mL. The common cause for urine output increase is excessive perioperative fluid load which leads to complications such as heart failure, pulmonary edema, and postoperative cognitive dysfunction (21) and the wide application of goal-oriented fluid therapy (GDFT) in clinical practice would effectively prevent it from happening. The partial dependence graphs showed that either a prolonged anesthesia duration or surgery duration would result in POD risk increase, with the latter’s partial dependence graph curve (the cutoff value at about 180 min) steeper than the former’s. The impact caused by anesthesia duration could be attributed to the inhibition of sedation and analgesic drugs on the central nervous system, whereas surgery trauma could increase the release of peripheral and central inflammatory factors and cause neuroinflammation and changes in cognitive function (22).

Among the postoperative influencing factors, the risk of POD would be minimized when the postoperative opioid dosage was less than 50 mg (converted to equivalent morphine dosage), but when the dosage was greater than 120 mg, the probability of POD occurrence increased significantly. In order to reduce the side effects of opioid overdose, general anesthesia combined with epidural or nerve block could be given preference during operation for its obvious advantages in perioperative cognitive improvement and POD prevention compared with simple general anesthesia (23). The increased incidence of POD caused by prolonged postoperative ICU treatment and tracheal intubation time could be explained by the ICU environment, stress state during tracheal intubation and the severity of patients’ disease *per se* (24). There is a clear correlation between the incidence of postoperative delirium and the degree of postoperative pain. Incomplete postoperative analgesia can enhance the patient’s stress response and alter the transmission of neurotransmitters. When postoperative analgesia is insufficient, patients may experience anxiety, irritability, resistance to communication, decreased motor function, slow recovery of gastrointestinal function, and changes in sleep cycle, all of which are factors leading to the occurrence of POD. Studies suggested that postoperative pain management may help reduce the risk of postoperative delirium in the elderly patients (25). In importance ranking, the maximum VAS value of exercise pain within 7 days after surgery, as a characteristic variable, ranks second in contribution to the predictive variable. In addition, this study showed that the median and maximum VAS values of resting pain and exercise pain within 7 days after surgery were the most closely correlated with POD occurrence, and when the pain was controlled within the mild range, the risk of POD was lowered, the risk rising with VAS values. Finally, as reported by Bilotta et al. (26), type of surgery was strong predictor of POD and for some surgical procedures-including orthopedic, abdominal aortic aneurysm, and cardiac thoracic surgery-it links to an increased risk. Compared between cardiac surgery and non-cardiac surgeries, the Odd Ratio of predictors for POD was: 3.5 (1.6–7.4). Therefore, we focus only on the non-cardiac thoracic and abdominal surgeries to reduce the influence of POD incidence by surgery type.

For classification of the imbalanced data in this study caused by the extremely low positive sample number in the data set, the cross validation and Smote technique (Synthetic minority over-sampling technique, Smote) were used to balance the data set and ensure excellent classification results in minority classes during model sampling, via retaining the majority class units and synthesizing new minority class units linearly from those that were set close (27, 28). In RF modeling, the selected ensemble algorithms adopted the data classification strategy of constructing multiple weaker classifiers, combining them into classifiers with strong classifier generalization performance, and forcing the classifiers to focus on minority class samples in the algorithmic level, which is advantageous over the regular approach of establishing a single strong classifier with excellent generalization ability in the training set in terms of unbalanced data modeling (29, 30). Besides, accuracy was not used as the single evaluation indicator in this study, because the overall accuracy of the imbalanced data classification would not accurately reflect the classification situation in minority classes. Instead, confusion matrix parameters (accuracy, AUC-ROC, AUC-PRC, and MMC scores) were adopted to comprehensively evaluate the model (31).

In this study, Boruta was used to screen and include 16 characteristic variables into the prediction model RF where importance ranking and univariate partial dependence graph were made to enhance its intelligibility, visibility, and potential applicability in clinical practice (32). Boruta algorithm generated “shadow attribute” for each variable and calculated the *Z*-score value for each of them through RF model. When the *Z*-score value was significantly higher than the highest shadow attribute value, the input variable was viewed and retained as dependent variable related one (33). Boruta follows all relevant feature selection methods and can capture all features related to the result variable. In contrast, most traditional feature selection algorithms follow a minimum optimization method, relying on a small subset of features and resulting in minimal errors in selecting classification. This method minimizes the error of the model to the greatest extent possible, which will ultimately form a minimum optimal feature subset. This occurs by selecting an overly condensed version of the input dataset, which in turn may result in the loss of some relevant features. On the other hand, Boruta finds all features, regardless of their correlation with the decision variable. This makes it very suitable for application in the field of biomedicine. In this article, POD related risk factors were screened and identified using Boruta, offering guidance for clinicians to take timely intervention measures for high-risk patients and reduce POD occurrence.

The challenges of applying machine learning lie primarily in the lack of interpretability and repeatability of machine learning-generated results, which may limit their application. Interpretable machine learning can effectively open the “black box” of machine learning (32, 34). In this study, the degree of contribution of each feature variable was explained through an importance sorting chart, and the trend of the result variable changing with the feature variable was explained through a univariate partial dependency profile and visualization prediction of random individual samples through a breakdown profile. This solves the problem of lack of interpretability in predictive models.

The following are the weaknesses of the present study that may have affected our results. Firstly, we included multiple risk factors, but did not include laboratory data. Secondly, POD subtypes can be divided into low, high, and mixed types, which we will continue to explore in subsequent studies. Thirdly, this article uses SMOTE technique to process imbalanced datasets, improving model performance while also potentially generating noise. Finally, this model requires an independent dataset to test its extrapolation and generalization capabilities. In the future, we will collect sufficient external validation datasets to further improve this model.

In this study, the major risk factors for the development of postoperative delirium are CREA and VAS-Move-Max. Machine learning algorithm can be established to predict the occurrence of postoperative delirium for older patients who underwent non-cardiac thoracic or abdominal surgery with general anesthesia.

## Data availability statement

The original contributions presented in the study are included in the article/Supplementary material, further inquiries can be directed to the corresponding authors.

## Ethics statement

The studies involving humans were approved by The trial protocol was approved by the Institutional Review Committee of Peking University. The studies were conducted in accordance with the local legislation and institutional requirements. The participants provided their written informed consent to participate in this study.

## Author contributions

WS: Writing – original draft, Formal analysis. XT: Formal analysis, Writing – review & editing. XH: Writing – review & editing, Data curation. PL: Data curation, Writing – review & editing. LL: Writing – review & editing, Formal analysis. HM: Funding acquisition, Writing – original draft. DW: Supervision, Writing – review & editing. TL: Supervision, Writing – review & editing.
