# Supplementary material for: Random forest algorithm for predicting postoperative delirium in older patients
Source: Front Neurol. 2024 Jan 11;14:1325941. doi: 10.3389/fneur.2023.1325941 (PMC10808713; doi:10.3389/fneur.2023.1325941)
Supplement: Supplementary file 1 [file Table_1.DOCX]

**Supplementary table**

**Random Forest Algorithm for Predicting Postoperative Delirium in Older Patients**

Weixuan Sheng^#,1^, Huihui Miao^#,1^, Xianshi Tang^2^, Xiaoyun Hu^1^, Pengfei Liu^1^, Lei Liu^3^, Dongxin Wang^*,4^, Tianzuo Li^*,1^

Supplementary Table 1. Baseline data

| Factor | | Non-POD | POD | *P-value* |
| --- | --- | --- | --- | --- |
| N | | 1662 | 58 |  |
| Age (years) | | 69 [64, 74] | 71 [66, 75] | 0.058 |
| Education (years) | | 9 [6, 13] | 8 [5, 13.] | 0.092 |
| BMI (kg/m^2^) | | 23.6 [21.5, 25.8] | 23.6 [21.1, 27.1] | 0.666 |
| MMSE score | | 29 [27, 30] | 29 [26, 30] | 0.858 |
| Anxiety score | | 0 [0, 2] | 0 [0, 2] | 0.839 |
| Depression score | | 0 [0, 2] | 2 [0, 4] | 0.001 |
| CHARLSON score | | 101 [50, 121] | 76 [50, 120] | 0.223 |
| Hct (%) | | 38.6 [35.4, 41.8] | 37.1 [34.5, 41.5] | 0.158 |
| ALB (g/L) | | 40.7 [37.4, 43.1] | 39.1 [36.3, 42.6] | 0.077 |
| GLU (mmol/L) | | 5.4 [4.9, 6.2] | 5.5 [4.9, 6.2] | 0.387 |
| Serum Na (mmol/L) | | 141.9 [140.0, 143.0] | 141.1 [139.2, 143.0] | 0.183 |
| Serum K (mmol/L) | | 4.0 [3.8, 4.3] | 4.1 [3.8, 4.4] | 0.191 |
| CREA (μmol/L) | | 86.0 [76.0, 99.0] | 73.5 [61.0, 93.0] | <0.001 |
| BUN (mmol/L) | | 5.6 [4.6, 6.8] | 5.5 [4.1, 6.1] | 0.058 |
| Nitrousoxide (%） | | 1.0 [0.0, 2.0] | 0.0 [0.0, 1.0] | <0.001 |
| Sevoflurance (%） | | 0.5 [0.0, 1.0] | 1.0 [0.5, 1.0] | <0.001 |
| Midazolam (mg) | | 1.5 [1.0, 2.0] | 2.0 [1.5, 2.0] | 0.001 |
| Urine (ml) | | 400 [100, 600] | 700 [325, 1000] | <0.001 |
| Bleeding (ml) | | 100 [50, 300] | 350 [100, 500] | <0.001 |
| MAP (mmHg） | | 80 [75, 86] | 82 [78, 89] | 0.013 |
| MHR (times/min) | | 66 [61, 73] | 72 [63, 80] | <0.001 |
| Anesthesia duration (min) | | 286 [221, 361] | 297 [236, 391] | 0.23 |
| Operation duration (min) | | 229 [166, 302] | 236 [185, 344] | 0.146 |
| Perioperative morphine (mg) | | 183.0 [146.0, 250.0] | 222.6 [164.0, 370.4] | 0.001 |
| Postoperative morphine (mg) | | 55.0 [50.0, 120.0] | 50.0 [50.0, 73.0] | 0.008 |
| ICU duration (min) | | 0 [0, 0] | 0 [0, 19] | <0.001 |
| Intubation duration (min) | | 0 [0, 0] | 0 [0, 1] | <0.001 |
| APACHE-II | | 0 [0, 0] | 0 [0, 8] | 0.005 |
| VAS-Rest-M | | 0 [0, 1] | 1 [0, 2] | <0.001 |
| VAS-Move-M | | 2 [1,4 ] | 3 [2, 4] | <0.001 |
| VAS-Rest-Max | | 2 [0, 3] | 2 [1, 3] | 0.008 |
| VAS-Move-Max | | 4 [2, 5] | 5 [4, 6] | <0.001 |
| VAS-Rest-Min | | 0 [0, 0] | 1 [0, 1] | <0.001 |
| VAS-Move-Min | | 1 [0, 2] | 2 [1, 3] | <0.001 |
| Gender | Male | 1089 (65.5) | 34 (58.6) | 0.345 |
|  | Female | 573 (34.5) | 24 (41.4) |  |
| ASA | ASA-I | 121 (7.3) | 2 (3.4) | 0.511 |
|  | ASA-II | 1421 (85.5) | 51 (87.9) |  |
|  | ASA-III | 120 (7.2) | 5 (8.6) |  |
| Group | GEA-PCEA | 842 (50.7) | 15 (25.9) | <0.001 |
|  | GA-PCIA | 820 (49.3) | 43 (74.1) |  |
| Stroke | Yes | 80 (4.8) | 5 (8.6) | 0.314 |
|  | No | 1582 (95.2) | 53 (91.4) |  |
| TIA | Yes | 22 (1.3) | 1 (1.7) | >0.999 |
|  | No | 1640 (98.7) | 57 (98.3) |  |
| COPD | Yes | 32 (1.9) | 0 (0.0) | 0.567 |
|  | No | 1630 (98.1) | 58 (100.0) |  |
| Chronic bronchitis | Yes | 30 (1.8) | 2 (3.4) | 0.677 |
|  | No | 1632 (98.2) | 56 (96.6) |  |
| Asthma | Yes | 24 (1.4) | 3 (5.2) | 0.088 |
|  | No | 1638 (98.6) | 55 (94.8) |  |
| Smoke | Yes | 398 (23.9) | 18 (31.0) | 0.279 |
|  | No | 1264 (76.1) | 40 (69.0) |  |
| CHD | Yes | 161 (9.7) | 5 (8.6) | 0.965 |
|  | No | 1501 (90.3) | 53 (91.4) |  |
| HT | Yes | 683 (41.1) | 28 (48.3) | 0.339 |
|  | No | 979 (58.9) | 30 (51.7) |  |
| Arrhythmia | Yes | 61 (3.7) | 2 (3.4) | >0.999 |
|  | No | 1601 (96.3) | 56 (96.6) |  |
| NYHA | I | 1261 (75.9) | 38 (65.5) | 0.099 |
|  | II | 401 (24.1) | 20 (34.5) |  |
| DM | Yes | 300 (18.1) | 14 (24.1) | 0.314 |
|  | No | 1362 (81.9) | 44 (75.9) |  |
| Thyroid diseases | Yes | 44 (2.6) | 1 (1.7) | 0.988 |
|  | No | 1618 (97.4) | 57 (98.3) |  |
| Liver  dysfunction | Yes | 14 (0.8) | 1 (1.7) | >0.999 |
|  | No | 1648 (99.2) | 57 (98.3) |  |
| HLP | Yes | 43 (2.6) | 3 (5.2) | 0.432 |
|  | No | 1619 (97.4) | 55 (94.8) |  |
| Renal  dysfunction | Yes | 6 (0.4) | 1 (1.7) | 0.58 |
|  | No | 1656 (99.6) | 57 (98.3) |  |
| Drink | Yes | 413 (24.8) | 13 (22.4) | 0.789 |
|  | No | 1249 (75.2) | 45 (77.6) |  |
| Atropine use | Yes | 1241 (74.7) | 43 (74.1) | >0.999 |
|  | No | 421 (25.3) | 15 (25.9) |  |
| Anti-nausea use | Yes | 1472 (88.6) | 56 (96.6) | 0.092 |
|  | No | 190 (11.4) | 2 (3.4) |  |
| NSAIDs use | Yes | 365 (22.0) | 6 (10.3) | 0.051 |
|  | No | 1297 (78.0) | 52 (89.7) |  |
| Intraoperative  hypotension | Yes | 963 (57.9) | 48 (82.8) | <0.001 |
|  | No | 699 (42.1) | 10 (17.2) |  |
| Postoperative  hypotension | Yes | 1596 (96.0) | 53 (91.4) | 0.157 |
|  | No | 66 (4.0) | 5 (8.6) |  |
